# Perinatal mortality in pregnancies with omphalocele: data from the Chinese national birth defects monitoring network, 1996–2006

**DOI:** 10.1186/1471-2431-14-160

**Published:** 2014-06-23

**Authors:** Kui Deng, Jie Qiu, Li Dai, Ling Yi, Changfei Deng, Yi Mu, Jun Zhu

**Affiliations:** 1 National Center for Birth Defects monitoring of China, West China Second University Hospital, Sichuan University, 17, Section3, Ren Min South Road, Chengdu, China; 2 Key Laboratory of Birth Defects and Related Diseases of Women and Children (Sichuan University), Ministry of Education, Chengdu, China; 3 Department of Maternal and Children Health, National Health and Family Planning Commission of the People's Republic of China, Beijing, China

**Keywords:** Omphalocele, Abdominal wall defects, Mortality, Perinatal outcome, Associated anomalies, Prenatal diagnosis, Ultrasound

## Abstract

**Background:**

Previous studies on the mortality rate of omphalocele are limited. The risk of death of non-isolated omphalocele and that of cases of omphalocele that are diagnosed prenatally by ultrasound are unclear. This study aimed to estimate the perinatal mortality of pregnancies with omphalocele. This study also examined the potential risk of death of non-isolated omphalocele and that of cases that are prenatally diagnosed by ultrasound.

**Methods:**

Data were retrieved from the national birth defects registry in China, for 1996–2006. Multinomial logistic regression was used to calculate the adjusted odds ratios (AORs) and 95% confidence intervals (CIs) between perinatal mortality and selected maternal and fetal characteristics.

**Results:**

Among 827 cases of omphalocele, 309 (37.4%) cases resulted in termination of pregnancy and stillbirth, and 124 (15.0%) cases resulted in death in the first 7 days after delivery, yielding a perinatal mortality rate of 52.4% (95% CI: 49.0–55.8%). The late fetal death rate (LFDR) of omphalocele that was diagnosed prenatally by ultrasound was 15.91-fold (AOR: 15.91, 95% CI: 10.18–24.87) higher than that of postnatally diagnosed cases. The LFDR of non-isolated omphalocele was 2.64-fold (AOR: 2.64, 95% CI: 1.62–4.29) higher than that of isolated cases. For the early neonatal death rate, neonates with non-isolated omphalocele had a 2.96-fold (AOR: 2.96, 95% CI: 1.82–4.81) higher risk than isolated cases, but the difference between prenatal ultrasound diagnosis and postnatal diagnosis was not significant.

**Conclusions:**

Selected fetal characteristics are significantly associated with the perinatal risk of death from omphalocele. Our findings suggest that improving pregnancy and delivery care, as well as management for omphalocele are important.

## Background

Omphalocele is among the more common anterior abdominal wall defects, and it is characterized as the absence of abdominal muscles, fascia, and skin. With omphalocele, there is herniation of the abdominal contents into the base of the umbilical cord, and these contents are covered by a membranous sac consisting of peritoneum and amnion [1,2]. Previous studies have estimated that the overall prevalence of omphalocele ranges from 2 to 3 per 10,000 births worldwide [3-10]. Omphalocele is also associated with a substantial risk of infant morbidity and mortality, which is a severe disadvantage to the short- and long-term life of affected newborns. Early surgical repair can improve the prognosis and increase the survival rate of omphalocele.

Previous reports regarding the mortality rate of omphalocele are limited. Few studies have reported the mortality of omphalocele using data from a large general population based on congenital malformation registries and national/regional epidemiological surveys. More recent studies have estimated the neonatal mortality of omphalocele using prenatal and neonatal databases from certain hospitals. However, these databases were confined to one hospital with a small number of cases, and the reported estimates with a wide range did not truly present the risk of death from omphalocele in the general population [11-14].

Several risk factors are associated with the perinatal outcome of pregnancies with omphalocele. As reported in most studies, omphalocele of concurrent with chromosomal anomalies or other structural malformations is more likely to be terminated electively and the fetus dies *in utero*[14-17]. Routine prenatal ultrasound screening allows identification of the majority of omphalocele early in gestation, but a high proportion of prenatally diagnosed cases of omphalocele end with termination of pregnancy or intrauterine death [3,14,18-20]. Previous studies did not independently investigate the risk of death from omphalocele with associated malformations using prenatal diagnosis by ultrasound because of potential confounding effects. The magnitude of effect estimates for the risk of death from non-isolated omphalocele and prenatally diagnosed cases by ultrasound has been imprecisely assessed.

This study aimed to estimate the perinatal mortality of omphalocele using consecutive data for 11 years from the national birth defects registry. We also aimed to examine the potential risk of death of fetuses and neonates with omphalocele by comparing the outcomes from different groups (non-isolated vs isolated groups and prenatal ultrasound diagnosis group vs postnatal diagnosis group).

## Methods

### Ascertainment of cases

Data on cases with omphalocele were obtained from the Chinese Birth Defects Monitoring Network (CBDMN) from January 1996 to September 2006. This network is a nationwide and hospital-based birth defects surveillance network covering a total of 450–471 hospitals (county level, city level, and provincial level) in China. The number of monitored births accounted for approximately 8–10% of the annual births in China [21]. The CBDMN used the passive case ascertainment method to identify congenital malformations, including live births, stillbirths, and termination of pregnancy in the member hospitals. The surveillance period for each abnormality was from 28 weeks of gestations to the first 7 days after birth. These cases were recognized by physical examination by trained obstetric and pediatric clinicians. The cases were confirmed by documentation of the postnatal diagnosis and narrative descriptions of abnormalities in the medical records. Cases diagnosed by prenatal ultrasonography were also confirmed by the postnatal records after delivery. A trained midwife was then asked to complete the “Birth Defects Register Form” registry and conduct online reporting quarterly, after which CBDMN staff reviewed all of these forms again. Incomplete forms and a nonspecific diagnosis were controlled by the midwives within 7 days to correct the final information. Pregnancies ending in stillbirth or elective termination, including an autopsy report where available, were reviewed to confirm or amend the final diagnosis. Written informed consent was obtained from the parents of neonates before they were discharged from the hospital. The consent mainly included the aims and importance of monitoring birth defects. This study was approved by the Ethic Review Committee of Sichuan University.

According to the International Clearinghouse for Birth Defects Monitoring Systems, omphalocele was defined as a midline abdominal wall defect, which was limited to an open umbilical cord. The viscera herniates into the base of the umbilical cord and is surrounded by the peritoneum and amniotic membranes [22]. The International Classification of Diseases, Tenth Revision, was used to code the diagnosis for omphalocele (Q79.2) in the national birth defects registry of China.

Permission was authored by National Health and Family Planning Commission to access data from the CBDMN. Data for this analysis were extracted based on the diagnosis code from the national birth defects registry that was developed by the CBDMN. The following variables were considered in our analysis: geographical location, maternal residence, maternal age, maternal education, gestational age, birthweight, presence or absence of other anomalies, prenatal or postnatal diagnosis, and the year of birth. Geographical location was divided into coastal areas, inland areas, and remote areas. Maternal age was categorized as 20–24 years old, 25–29 years old, 30–34 years old, and 35–39 years old. Residence referred to that of the mothers, and was divided into rural (countries or suburban areas) and urban (towns or cities) areas. Gestational age was divided into 28–36 weeks and 37–42 weeks. Birthweight was grouped into <2500 g and ≥2500 g. Isolated omphalocele was defined as omphalocele, which occurred without chromosomal or structural malformations. Non-isolated omphalocele was defined as cases with associated chromosomal or structural malformations, which were not related to omphalocele. Prenatal diagnosis refers to cases of omphalocele that were detected prenatally by ultrasound. Postnatal diagnosis refers to cases of omphalocele that were detected by physical examination after birth. The response variable was perinatal mortality of birth with omphalocele, which was categorized into late fetal death (LFD) and early neonatal death (ENND). LFD was defined as the death of a fetus later than the gestational age of 28 weeks. ENND was defined as the death of a neonate in the first 7 days of life. Data on the mortality of affected pregnancies were also extracted from the national birth defects registry.

### Data quality management

Data quality management (DQM) was routinely evaluated for surveillance data. The DQM teams consisted of five upper-level CBDMN experts. These experts verified data collection, data reporting, diagnosis of defects, and obstetric and pediatric medical records according to the surveillance manual. This was performed to improve the accuracy, comparability, completeness, and timeliness of the registered data. For DQM at the county level, all of the member hospitals were investigated quarterly. For DQM at the provincial and national levels, cluster sampling covered approximately one-third and 10% of the member hospitals, respectively. Provincial- and national-level DQM were conducted semi-annually and annually, respectively. The under-reporting rate of live births or malformations needed to be no greater than 1%, and errors or missing information on the report form had to be no greater than 1%. At each level, a panel of senior health professionals evaluated the completeness, accuracy, and timeliness of the data.

### Statistical analysis

The perinatal mortality rate of omphalocele was the sum of the late fetal death rate (LFDR) and early neonatal death rate (ENNDR). The LFDR was calculated by the number of stillbirths and termination of pregnancies divided by the total number of births with omphalocele. The ENNDR was calculated by the number of neonatal deaths within the first 7 days after birth divided by the total number of births with omphalocele.

The Cochran–Armitage trend test was used to assess the changes in mortality of omphalocele over time. Because the response variables in our analysis were nominal and for which there were two categories, multinomial logistic regression was used to generate the odds ratios and 95% confidence intervals (CIs) between the rate of LFD/ENND and selected maternal/fetal characteristics, while controlling for confounding factors to evaluate the effect of each variable. The estimated risks were adjusted by potential confounders, which were selected on the basis of the results of the bivariate analysis and previously reported evidence. All tests of hypotheses were two-tailed with a type 1 error rate fixed at 5%. Statistical analyses were performed using SAS 9.1 software (SAS Institute Inc., Cary, NC).

Since September 2006, the number of the member hospitals has almost doubled. Therefore, to ensure the comparability of registered data from the member hospitals, our study period was confined to January 1996 to September 2006. During the study period, because the number of births in some of the sampled surveillance hospitals had declined, we increased the number of hospitals that were selected from neighboring counties to monitor a sufficient amount of births to ensure a representative sampled population. Additionally, some of the member hospitals were replaced by other neighboring hospitals because of reorganization of their medical services. Therefore, the number of hospitals over the study period changed in our analysis.

## Results

From January 1996 to September 2006, a total of 827 cases of omphalocele were identified from the CBDMN, which included termination of pregnancy, stillbirths, and live births. Of these, 322 (39.3%) cases were diagnosed antenatally by ultrasound and 501 cases (60.6%) were confirmed by physical examination after birth. Four cases had missing diagnosis information. Among the 827 cases, isolated omphalocele occurred in 596 (72.1%) cases and 231 (27.9%) cases were non-isolated omphalocele. The most commonly associated anomaly with omphalocele was cleft lip with or without cleft palate or cleft palate (n = 45), followed by neural tube defects (n = 36), and then polydactyly or syndactyly (n = 33).

The perinatal mortality of pregnancies with omphalocele is shown in Table 1. A total of 309 fetuses died after 28 weeks’ gestation and 124 neonates were dead within the first 7 days after birth. The perinatal mortality rate, LFDR, and ENNDR of omphalocele was 52.4% (95% CI: 49.0–55.8%), 37.4% (95% CI: 34.1–40.7%), and 15.0% (95% CI: 12.6–17.4%), respectively. The risk of death of a fetus was nearly 2.5 times higher compared with that of a neonate.

**Table 1:** Perinatal mortality of pregnancies with omphalocele from 1996 to 2006, China

**Years**	**Total N**	**Late fetal death**		**Early neonatal death**		**Perinatal mortality**	
		**No.**	Rate* (95% CI)	**No.**	Rate* (95% CI)	**No.**	Rate* (95% CI)
1996	56	19	33.9 (21.5, 46.3)	15	26.8 (15.2, 38.4)	34	60.7 (47.9, 73.5)
1997	46	19	41.3 (27.1, 55.5)	8	17.4 (6.4, 28.3)	27	58.7 (44.5, 72.9)
1998	71	19	26.8 (16.5, 37.1)	9	12.7 (4.9, 20.4)	28	39.4 (28.1, 50.8)
1999	83	29	34.9 (24.7, 45.2)	10	12.1 (5.0, 19.1)	39	47.0 (36.3, 57.7)
2000	83	30	36.1 (25.8, 46.5)	15	18.1 (9.8, 26.4)	45	54.2 (43.5, 64.9)
2001	67	19	28.4 (17.6, 39.2)	11	16.4 (7.6, 25.3)	30	44.8 (32.9, 56.7)
2002	75	28	37.3 (26.4, 48.3)	12	16.0 (7.7, 24.3)	40	53.3 (42.0, 64.6)
2003	66	20	30.3 (19.2, 41.4)	16	24.2 (13.9, 34.6)	36	54.6 (42.5, 66.6)
2004	101	45	44.6 (34.9, 54.3)	5	5.0 (0.7, 9.2)	50	49.5 (39.8, 59.3)
2005	115	53	46.1 (37.0, 55.2)	16	13.9 (7.6, 20.2)	69	60.0 (51.1, 69.0)
2006	64	28	43.8 (31.6, 55.9)	7	10.9 (3.3, 18.6)	35	54.7 (42.5, 66.9)
Total	827	309	37.4 (34.1, 40.7)	124	15.0 (12.6, 17.4)	433	52.4 (49.0, 55.8)

*Rate is the number of death per 100 fetus and neonates with omphalocele from 28 weeks of gestations to the first 7 days of life.

CI, confidence interval.

Over the study period, there were annual fluctuations for the ENNDR. The highest rate was in 1996 (26.8%, 95% CI: 15.2–38.4%) and the lowest was in 2004 (5.0%, 95% CI: 0.7–9.2%). There was a significant difference in the annual ENNDR (p < 0.05) during the study period, but not for the LFDR (p > 0.05) and perinatal mortality rate (p > 0.05). Furthermore, an upward trend was observed for the LFDR but a downward trend was observed for the ENNDR for 1996–2006, using the Cochran-Armitage trend test (both p < 0.05). In contrast, no trend was shown for the perinatal mortality rate (p > 0.05).

Table 2 shows the association between the mortality of fetuses or neonates and selected maternal characteristics. Fetuses or neonates with omphalocele who were located in inland areas had a 1.56-fold or 1.72-fold higher mortality than those in coastal areas [adjusted odds ratio (AOR): 1.56, 95% CI: 1.09–2.24; AOR: 1.72, 95% CI: 1.03–2.85, respectively]. However, there was no significant difference in mortality for the LFDR or ENNDR in remote areas compared with coastal areas (AOR: 1.07, 95% CI: 0.73–1.57; AOR: 1.53, 95% CI: 0.91–2.59, respectively). Neonates born to mothers with primary school or unschooled education had a 2.76-fold higher ENNDR compared with those born to mothers who had gone to high school (AOR: 2.76, 95% CI: 1.35–5.63), whereas this phenomenon did not occur with the LFDR (AOR: 0.89, 95% CI: 0.51–1.56). The LFDR and ENNDR of neonates born to mothers with more than high school education were not significantly different from those born to mothers with high school education (AOR: 0.97, 95% CI: 0.61–1.54; AOR: 0.97, 95% CI:0.45–2.09, respectively). Neonates of women who resided in rural areas had a higher risk of LFD or ENND than those of women in urban areas, but this difference was not significant (AOR: 1.34, 95% CI: 0.91–1.97; AOR: 1.32, 95% CI: 0.81–2.18, respectively). Similarly, the mortality of fetuses or neonates born to women in the lower maternal age groups had a higher risk of death compared with those born to women in the higher maternal age groups, but this difference was not significant.

**Table 2:** Association with perinatal death of omphaloceles by the selected maternal characteristics, China, 1996-2006

**Characteristics**	**Total N**	**Late fetal death**				**Early neonatal death**			
		**No.**	Rate* (95% CI)	**COR (95% CI)**	**AOR**^**† **^**(95% CI)**	**No.**	Rate* (95% CI)	**COR (95% CI)**	**AOR**^**† **^**(95% CI)**
**Geographical location**									
Coastal areas	308	108	35.1 (29.7, 40.4)	Ref.	Ref.	37	12.0 (8.4, 15.6)	Ref.	Ref.
Inland areas	285	121	42.5 (36.7, 48.2)	1.55 (1.09, 2.20)	1.56 (1.09, 2.24)	46	16.1 (11.9, 20.4)	1.72 (1.05, 2.81)	1.72 (1.03, 2.85)
Remote areas	234	80	34.2 (28.1, 40.3)	1.07 (0.73, 1.56)	1.07 (0.73, 1.57)	41	17.5 (12.7, 22.4)	1.60 (0.97, 2.65)	1.53 (0.91, 2.59)
**Maternal residence**									
Urban	518	190	36.7 (32.5, 40.8)	Ref.	Ref.	63	12.2 (9.3, 15.0)	Ref.	Ref.
Rural	309	119	38.5 (33.1, 43.9)	1.29 (0.94, 1.76)	1.34 (0.91, 1.97)	61	19.7 (15.3, 24.2)	1.99 (1.32, 3.00)	1.32 (0.81, 2.18)
**Maternal age**^$^**(years)**									
20–24	277	104	37.5 (31.8, 43.2)	1.12 (0.79, 1.58)	1.05 (0.73, 1.52)	54	19.5 (14.8, 24.2)	1.57 (1.00, 2.46)	1.16 (0.72, 1.88)
25–29	358	135	37.7 (32.7, 42.7)	Ref.	Ref.	50	14.0 (10.4, 17.6)	Ref.	Ref.
30–34	131	47	35.9 (27.7, 44.1)	0.87 (0.57, 1.35)	0.90 (0.58, 1.41)	15	11.5 (6.0, 16.9)	0.75 (0.40, 1.43)	0.70 (0.36, 1.35)
35–39	59	22	37.3 (24.9, 49.6)	0.88 (0.49, 1.59)	0.90 (0.50, 1.63)	5	8.5 (1.4, 15.6)	0.54 (0.20, 1.46)	0.50 (0.18, 1.37)
**Maternal education**^#^									
Primary school/unschooled	121	40	33.1 (24.7, 41.4)	1.07 (0.65, 1.76)	0.89 (0.51, 1.56)	32	26.4 (18.6, 34.3)	3.35 (1.78, 6.30)	2.76 (1.35, 5.63)
Junior school	337	125	37.1 (31.9, 42.2)	1.06 (0.74, 1.52)	0.93 (0.62, 1.39)	57	16.9 (12.9, 20.9)	1.89 (1.10, 3.24)	1.66 (0.93, 2.99)
High school	231	90	39.0 (32.7, 45.2)	Ref.	Ref.	23	10.0 (6.1, 13.8)	Ref.	Ref.
More than high school	131	50	38.2 (29.8, 46.5)	0.95 (0.60, 1.50)	0.97 (0.61, 1.54)	12	9.2 (4.2, 14.1)	0.89 (0.42, 1.91)	0.97 (0.45, 2.09)

^*^Rate is the number of death per 100 fetus and neonates with omphalocele from 28 weeks of gestations to the first 7 days of life.

^**†**^ORs were adjusted by geographical location, maternal residence, maternal age, and maternal education.

^$^Two cases with unregistered maternal age were excluded in this analysis. Group of <20 years was combined into group of 20–24 years and group of ≥40 years was also combined into group of 35–39 years because of the small number of cases in these groups.

^#^Seven cases with unknown maternal education were excluded in this analysis.

COR, crude odds ratio; AOR, adjusted odds ratio; CI, confidence interval; Ref., reference group.

Table 3 shows the association between the mortality of omphalocele-affected pregnancies and selected fetal characteristics. The LFDR at the gestational ages of 28–36 weeks was 2.42-fold higher than that of 37–42 gestational weeks (AOR: 2.42, 95% CI: 1.52–3.86). Additionally, birthweight of <2500 g was 3.17-fold higher, non-isolated omphalocele was 2.64-fold higher, and diagnosis by prenatal ultrasound was 15.91-fold higher compared with birthweight of ≥2500 g, isolated omphalocele, and diagnosis by postnatal ultrasound, respectively (AOR: 3.17, 95% CI: 1.97–5.09; AOR: 2.64, 95% CI: 1.62–4.29; AOR: 15.91, 95% CI: 10.18–24.87, respectively). The LFDR of omphalocele that was diagnosed by prenatal ultrasound was the highest, followed by <2500 g birthweight, and then non-isolated omphalocele. Neonates with <2500 g birthweight and non-isolated omphalocele had a higher ENNDR than that of the reference groups (AOR: 1.72, 95% CI: 1.04–2.82; AOR: 2.96, 95% CI: 1.82–4.81, respectively). The mortality of neonates who were born at 28–36 gestational weeks and the LFDR of cases of prenatally diagnosed omphalocele were slightly higher than those of the reference groups, but these differences were not significant (AOR: 1.18, 95% CI: 0.72–1.96; AOR: 1.42, 95% CI: 0.81–2.50, respectively). Neonates with omphalocele and non-isolated abnormalities had the highest rate of death in the first 7 days of life, followed by neonates who were <2500 g birthweight at birth.

**Table 3:** Association with perinatal mortality of omphaloceles by the selected fetal characteristics, China, 1996-2006

	**Total N**	**Late fetal death**				**Early neonatal death**			
		**No.**	Rate* (95% CI)	**COR (95% CI)**	**AOR**^**† **^**(95% CI)**	**No.**	Rate * (95% CI)	**COR (95% CI)**	**AOR**^**† **^**(95% CI)**
**Gestational age**^**$ **^**(weeks)**									
37–42	436	83	19.0 (15.4, 22.7)	Ref.	Ref.	74	17.0 (13.4, 20.5)	Ref.	Ref.
28–36	388	224	57.7 (52.8, 62.6)	6.61 (4.73, 9.22)	2.42 (1.52, 3.86)	50	12.9 (9.6, 16.2)	1.65 (1.09, 2.52)	1.18 (0.72, 1.96)
**Birthweight**^#^** (g)**									
≥2500	428	80	18.7 (15.0, 22.4)	Ref.	Ref.	65	15.2 (11.8, 18.6)	Ref.	Ref.
<2500	395	226	57.2 (52.3, 62.1)	7.20 (5.15, 10.83)	3.17 (1.97, 5.09)	58	14.7 (11.2, 18.2)	2.28 (1.50, 3.45)	1.72 (1.04, 2.82)
**Non-isolated omphalocele**									
No	596	198	33.2 (29.4, 37.0)	Ref.	Ref.	76	12.8 (10.1, 15.4)	Ref.	Ref.
Yes	231	111	48.1 (41.6, 54.5)	2.51 (1.78, 3.54)	2.64 (1.62, 4.29)	48	20.8 (15.5, 26.0)	2.82 (1.81, 4.40)	2.96 (1.82, 4.81)
**Prenatal ultrasound diagnosis**^§^									
No	498	70	14.1 (11.0, 17.1)	Ref.	Ref.	99	19.9 (16.4, 23.4)	Ref.	Ref.
Yes	322	235	73.0 (68.1, 77.8)	17.81 (12.17, 26.06)	15.91 (10.18, 24.87)	25	7.8 (4.8, 10.7)	1.34 (0.80, 2.25)	1.42 (0.81, 2.50)

^*^Rate is the number of death per 100 fetus and neonates with omphalocele from 28 weeks of gestations to the first 7 days of life.

^**†**^ORs were adjusted by geographical location, maternal residence, maternal age, maternal education, gestational age, birthweight, non-isolated omphalocele and diagnosis by prenatal ultrasonography.

^$^Three cases with unknown gestation age were excluded from this analysis.

^#^Four cases with unknown birthweight were excluded from this analysis.

^§^Seven cases with unknown prenatal ultrasound diagnosis were excluded from this analysis.

COR, crude odds ratio; AOR, adjusted odds ratio; CI, confidence interval; Ref., reference group.

## Discussion

We found that the perinatal mortality of pregnancies with omphalocele was 52.4%, late fetal mortality was 37.4%, and early neonatal mortality was 15.0%. These estimates are in line with previous research showing that 39–41% of cases of omphalocele result in termination of pregnancy and stillbirth, and 12% of cases result in neonatal death [4,5]. However, higher estimates than our results have been reported in other birth defects registries [6,9,12,18,23,24]. The inconsistency in the mortality rate for omphalocele may be owing to the registered gestational weeks of pregnancy, prenatal detection, follow-up period, and prenatal and postnatal care, as well as management of omphalocele.

After controlling for confounding factors, we observed that prenatally diagnosed omphalocele was more likely to result in LFD compared with non-isolated omphalocele. This finding is supported by previous studies on the perinatal outcome of fetal omphalocele following prenatal diagnosis [12,14,20]. However, most of these previous results were mixed by the effect of non-isolated and prenatal diagnosis, showing that a high proportion of omphalocele was prenatally diagnosed by ultrasound, and fetuses were electively terminated or died *in utero*. If parents were properly counseled by a pediatrician and intrauterine transfer occurred to tertiary units with pediatric surgical facilities, the outcome of prenatally diagnosed omphalocele would be more favorable [13].

For neonates within 7 days old, those with non-isolated omphalocele had a higher risk of death than prenatally diagnosed cases. Up to 70% of omphalocele cases are associated with other structural malformations, chromosomal abnormalities, and genetic syndromes, and this phenomenon is significantly associated with the ultimate prognosis for these fetuses [15,25-27]. In addition, prenatal ultrasound diagnosis did not significantly increase the risk of death of a neonate in early life, with similar results found in another study [20]. This finding can be explained by the following two points. First, prenatal ultrasonography has become a routine examination in pregnancy. The sensitivity of prenatal ultrasound screening in detecting omphalocele is 75% in the second trimester of pregnancy, ranking second among all of the congenital malformations that are diagnosed prenatally by ultrasound (anencephaly is the first) [24,28]. Second, prenatally diagnosed cases include more fetuses with a giant omphalocele or liver herniation compared with those postnatally diagnosed, and most women opt for termination of pregnancy or intrauterine death occurs [20].

There are several limitations to our study. First, we could not distinguish between termination of pregnancy from stillbirth in our analysis. Therefore, we could not estimate the proportion of electively terminated pregnancies and the proportion of stillbirths. Second, our monitoring period covered the period from 28 gestational weeks to the first 7 days after delivery. This means that we did not investigate the death of fetuses before the age of 28 gestational weeks. Consequently, our reported mortality may underestimate the true risk of death from omphalocele. Third, because some women who had fetuses with omphalocele terminated pregnancy or death occurred *in utero*, they refused an autopsy. Therefore, the characteristics of these cases could not be verified postnatally, resulting in the misclassification of other associated anomalies as isolated omphalocele and the misclassification of gastroschisis and other abdominal wall defects as omphalocele. Finally, because this study was hospital-based and it focused on selected hospitals rather than all deliveries in a region, the hospital-based samples may have introduced referral bias. However, because of the wide geographic coverage, consistent case ascertainment, and the large sample size, the CBDMN data used in our study were reliable.

## Conclusions

Our findings contribute to the growing body of estimates regarding perinatal mortality in fetuses and neonates with omphalocele. Cases of prenatally diagnosed omphalocele have a higher risk of LFD, while there is no significant risk of death for neonates with omphalocele when they are diagnosed prenatally. Those with non-isolated omphalocele are more likely to die in the early neonatal period. Improving pregnancy and delivery care, as well as management for omphalocele are important. Further studies are needed to include more current data to investigate the perinatal mortality of pregnancies with omphalocele.

## Abbreviations

CBDMN: Chinese birth defects monitoring network; LFD: Late fetal death; ENND: Early neonatal death rate; DQM: Data quality management; LFDR: Late fetal death rate; ENNDR: Early neonatal death rate; COR: Crude odds ratio; AOR: Adjusted odds ratio; CI: Confidence interval.

## Competing interests

The authors declare that they have no competing interests.

## Authors’ contributions

DK and QJ were joint first authors and participated equally in the study design, literature review, data analysis, manuscript writing, and final revision of the article; DL, YL, DCF, and MY participated in the acquisition of data and the interpretation of data; ZJ participated in the study design, coordination and critical revision of the manuscript. All authors read and approved the final manuscript.

## Pre-publication history

The pre-publication history for this paper can be accessed here:

http://www.biomedcentral.com/1471-2431/14/160/prepub
